# A Cell-Based Method to Detect Agonist and Antagonist Activities of Endocrine-Disrupting Chemicals on GPER

**DOI:** 10.3389/fendo.2020.00547

**Published:** 2020-08-14

**Authors:** Séverine Périan, Catherine Cerutti, Christelle Forcet, Violaine Tribollet, Jean-Marc Vanacker

**Affiliations:** Institut de Génomique Fonctionnelle de Lyon, Université de Lyon, Université Lyon 1, CNRS UMR5242, Ecole Normale Supérieure de Lyon, Lyon, France

**Keywords:** GPER, endocrine-disrupting chemicals, pharmacology, screening, fibroblasts

## Abstract

Endocrine-disrupting chemicals (EDCs) are exogenous compounds that impact endogenous hormonal systems, resulting in adverse health effects. These chemicals can exert their actions by interfering with several pathways. Simple biological systems to determine whether EDCs act positively or negatively on a given receptor are often lacking. Here we describe a low-to-middle throughput method to screen the agonist/antagonist potential of EDCs specifically on the GPER membrane estrogen receptor. Application of this assay to 23 candidate EDCs from different chemical families reveals the existence of six agonists and six antagonists.

## Introduction

Endocrine-disrupting chemicals (EDCs) can be defined as exogenous compounds that can interfere with hormonal signaling (1). Chemicals classified as EDCs are produced for various industrial purposes (for example as components of pesticides, cosmetic products or plastic components) and belong to different chemical families (for example alkylphenols, parabens or phthalates). EDCs can accumulate in the environment (with varying levels of persistence), diet as well as body fluids and tissues. The adverse health effects elicited by EDCs can be diverse. For instance, as evaluated by epidemiological studies and/or experimental set up, exposure to Bisphenol A (BPA, a prototypical EDC) is correlated with increased cancer risk, obesity and reproductive health defects (2–5). EDCs can impact diverse levels of endocrine signaling, ranging from hormone production to hormone receptor expression and downstream signaling. Mechanistically, their action can be mediated by several receptors onto which they act as agonists or antagonists. For instance, BPA has been reported to dysregulate the activity of several nuclear receptors, such as the estrogen receptors (ERs), androgen receptor (AR) or estrogen-related receptor γ (ERRγ) [(6–11); reviewed in (12)]. EDC receptors are often co-expressed in given cells and tissues, complicating the mechanistic interpretation of the results. As a consequence, determining whether an EDC targets a given receptor leading to the dysregulation of discrete pathway(s), can be a laborious task. There is thus a need for simple systems in which a measurable effect can be directly ascribed to the (dys)regulation of a single receptor/pathway.

G protein-coupled estrogen receptor (GPER, previously known as GPR30 or GPER1) is a membrane-localized receptor with capacities to bind estrogens (13–15) and to crosstalk with the classical nuclear estrogen receptors (16, 17). GPER signaling is involved in various physiological and pathological processes such as metabolic regulations, diabetes and atherosclerosis, or cancer progression (18–21), suggesting that it could contribute, at least in part, to some of the adverse effect of EDCs. In support to this hypothesis, studies at the cellular level have shown that, in addition to endogenous estrogens, GPER activity can be modulated by several compounds including synthetic selective agonists or antagonists (e.g., G-1 or G-15 and G-36, respectively), selective estrogen receptor modulators (SERMs) and bisphenols (22–25). Altogether, this suggests a capacity of GPER to respond to a broad spectrum of chemicals, including EDCs, many of which remain unknown. This also points to the need of defining a simple test to easily identify agonists/antagonists of this pathway.

Our previous work (26) showed that primary human dermal fibroblasts (hDF), which do not express ER, display a quantifiable morphological change in response to 17β-estradiol (E2), in a strict GPER-dependent manner. This suggested that this cellular phenomenon could be used as a read-out of GPER activation. However, primary cultures originate from different donors. Thus, inter-individual variability in the response as well as a possible exhaustion of the cell batches, may jeopardize reproducibility and efficiency. Using the MRC5 human fibroblast cell line, we here report the establishment of a method for a low-to-middle throughput screen of compounds acting on GPER as agonist or antagonist. We apply this cell-based method to define the capacity (or lack thereof) of 23 EDCs from various chemical families to modulate GPER activity.

## Materials and Methods

### Cells

Cells were cultured in DMEM supplemented with 10% FCS, 10 U/ml penicillin and 10 μg/ml streptomycin (complete medium). For proliferation tests and evaluation of compound toxicity, 2 × 10^4^ MRC5 cells were seeded in 96-well plates and assessed for cell number using CellTiterGlo kit (Promega).

For cell shape studies using the Cytonote lens-free cell imaging device (Iprasense, Montpellier, France), 10,000 MRC5 cells were seeded in 400 μl of complete medium in 4-chambers culture dishes. After 24 h, medium was changed to 600 μl phenol red-free DMEM without serum and cells were further incubated for 48 h. Ten microliter phenol red-free medium containing the tested compound were then added and cell cultures were immediately analyzed for 3 or 4 h in the Cytonote system. ImageJ was used to analyze the reconstituted images of the cell cultures at time point 0, 60, 120, 180, and 240 min after compound addition. Except were indicated, 30 cells per experiment were individually followed at all these time points, at which the ratio long axis to short axis was measured. Suspected antagonists were added 15 min before agonists.

For immunofluorescence experiments, cells (40% confluent) were cultured on glass slides, fixed with 4% paraformaldehyde and then washed with PBS 1x. FITC-phalloidin (P5283, Sigma, 1/750) was then added for 1 h. Nuclei were counterstained with Hoescht staining. Pictures were taken with Zeiss-Axiovert and images were processed and analyzed with the open-source package ImageJ with custom plug-in routines.

### Compounds

All compounds used were resuspended in DMSO in 1,000x stock solutions. Characteristics and provenance of EDCs used in this study are shown on Table S5. 17β-estradiol (E2) was purchased from Sigma-Aldrich, G-1 from Cayman, G-15 and G-36 from Tocris.

### Expression Analyses

For siRNA transfection, 3 × 10^−5^ cells per ml were seeded in 6-well plate and 25 pmol/ml of siRNA were transfected with INTERFERin (Polyplus Transfection) according to the manufacturer's recommendations.

For Western blot analysis, cells were lysed in NP40 buffer supplemented with Protease Inhibitor Cocktail (Sigma Aldrich). Proteins (50 μg) were resolved on 10% SDS-PAGE, blotted onto PVDF membrane (GE-Healthcare) and probed with specific antibodies after saturation. Primary antibodies used in this study were: hsp90 (API-SPA-830, Enzo Life Sciences, 1/3,000), ERα (sc-8002 F-10 Santa Cruz, 1/1,000), and GPER (sc-48525-R, Santa Cruz, 1/500). Secondary antibodies were: anti-rabbit IgG for ERα and GPER, anti-mouse IgG for hsp90 (W4011 and W402B, respectively; Promega, 1/10,000).

Total RNAs were extracted by the guanidinium thiocyanate/phenol/chloroform method. One microgram of RNA was converted to first strand cDNA using the RevertAid kit (ThermoScientific). Real time PCRs were performed in 96-well plates using the IQ SYBR Green Supermix (BioRad). Data were quantified by ΔΔ-Ct method and normalized to 36b4 expression.

Sequences of the PCR primers used in this study:

36b4: 5′-GTCACTGTGCCAGCCCAGAA-3′ and 5′-TCAATGGTGCCCCTGGAGAT-3′

GPER: 5′-AGGGACAAGCTGAGGCTGTA-3′ and 5′-GTCTACACGGCACTGCTGAA-3′

Sequences of the siRNA used in this study:

GPER#1: 5′-GGCUGUACAUUGAGCAGAA-3′ and 5′-UUCUGCUCAAUGUACAGCC-3′

GPER#2: 5′-AGCUGAGGCUGUACAUUGA-3′ and 5′-UCAAUGUACAGCCUCAGCU-3′

### Statistical Analyses

#### Distribution of L/s Ratio Over Cells

Using data from the E2 (10^−7^ M) condition obtained on 244 cells, the L/s ratio data distribution was examined using the Shapiro-Wilk normality test. The distribution of L/s ratios observed at each time, as well as the distribution of L/s ratio differences between two exposure times for each cell were found significantly non normal (*p* < 10^−5^). In addition, homogeneity of variance was tested with the Levene's test. It showed that variance of L/s ratio differences between two times significantly differs (*p* < 10^−8^). Therefore, L/s ratio was summarized by its median over cells for each condition and only non-parametric statistical tests were used.

#### L/s Ratio Normalization

For each condition, individual L/s ratios were expressed as relative to the median value at exposure time 0 min.

#### Comparing L/s Ratios

The comparison of L/s ratios between more than 2 exposure times used the non-parametric Friedman test for repeated measures. *Post-hoc* tests between two times used the Wilcoxon signed-rank test with Bonferroni *p*-value correction. When considering L/s ratios for 2 exposure times, the Wilcoxon signed rank test was used. The comparison of L/s ratios between several conditions or experiments at exposure time 180 used the Kruskal-Wallis test followed by the Mann-Whitney test for comparison between 2 groups. Statistical significance was taken at *p* < 0.05.

## Results

**Figure 1:**
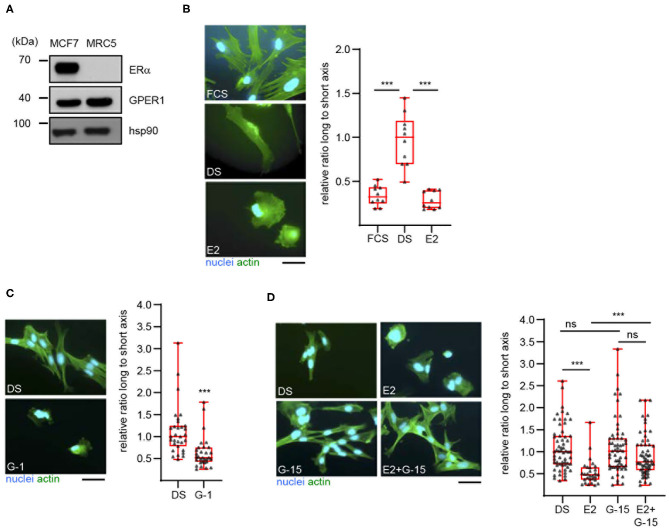
17β-estradiol induces a morphological change in MRC5 cells in a GPER-dependent manner. **(A)** Expression of the indicated proteins was determined in MCF7 and MRC5 cells by Western blot. Hsp90 was used as a loading control. Significance relative to time 0 (or between times 120 and 240, as indicated) was analyzed using Wilcoxon signed-rank test. ***p < 0.0005. **(B)** MRC5 cells were cultured in the presence of untreated (FCS), or desteroidated serum-containing medium supplemented with vehicle (DS) or 10^−7^ M 17β-estradiol (E2). **(C)** MRC5 cells were treated with 10^−7^ M G-1, a GPER agonist. **(D)** Cells were treated with 10^−8^ M E2 and/or 10^−7^ M G-15, a GPER antagonist. Actin and nuclei were stained. Ratio between the long and short cell axes was determined using ImageJ. Data are expressed relative to the median ratio under DS conditions. **(B)** *n* = 10; **(C)** *n* = 30; **(D)** *n* = 60. Significance was determined using Mann-Whitney test. ***p < 0.0005; ns: non significant. Scale bar: 50 μm.

To extend our previous findings to a model cell line, we examined the estrogenic response of MRC5 cells, an immortalized human fibroblast cell line. Western blot analysis first showed that these cells indeed express GPER, but not the classical estrogen receptor α (Figure 1A). As expected, both receptors were found expressed in the human mammary cell line MCF7. The effect of E2 on the morphology of MRC5 cells was next examined (Figure 1B). To this end, cells were exposed to culture medium containing untreated serum (FCS, Fetal Calf Serum) or desteroidated serum (DS) supplemented or not with E2. After fixation, actin was labeled to visualize cell morphology. The longest and shortest cell axes (L and s, respectively) were measured and cell shape was expressed as the ratio (L/s) between these axes. We noted a more elongated shape (i.e., high L/s ratio) in cells exposed to DS medium, as compared to FCS-exposed cells. Interestingly, E2 supplementation reversed this phenotype. It is unlikely that this phenomenon involves cell proliferation. Indeed, whereas proliferation of MRC5 cells was abolished in DS medium as compared to FCS one, E2 addition did not reverse this effect (Figure S1A).

These data suggest that E2 promotes MRC5 cell spreading in a GPER-dependent manner. To prove this dependence, we first searched to inactivate GPER in MRC5 cells, using siRNAs. However, these siRNAs were efficient at the RNA- but not at the protein level (Figure S1B), suggesting a high stability of GPER protein and preventing the use of siRNAs in our experiments. We thus turned to a pharmacological approach. We observed that exposure to G-1, a GPER synthetic agonist, efficiently reduces cell elongation (Figure 1C). We also used G-15, a GPER synthetic antagonist (Figure 1D). By itself, this compound is unable to induce any morphological change in MRC5. However, G-15 efficiently blocks the E2-induced cell spreading, indicating that GPER mediates this estrogenic response. Altogether, MRC5 cells display E2-responses that are similar to primary hDF and could thus be used to measure GPER activation without interference from ERα.

**Figure 2:**
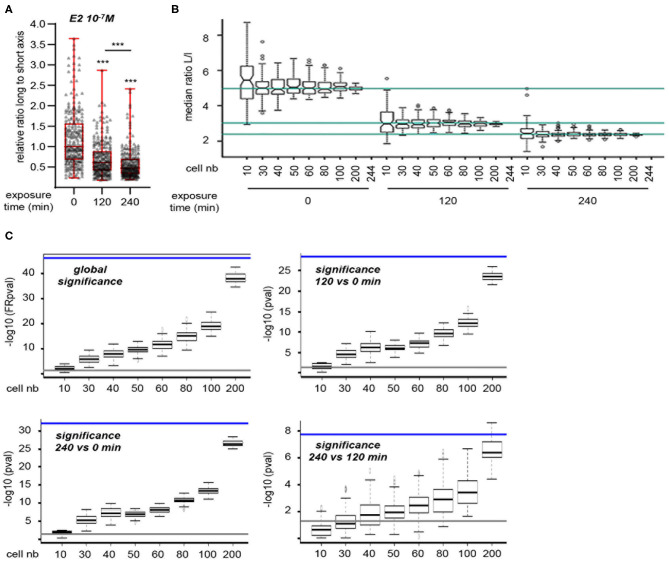
Statistical validation of the approach. **(A)** Cells were exposed to 10^−7^ M E2. 244 cells were individually analyzed for L/s ratio. **(B)** Medians of these ratios at 0, 120, and 240 min are indicated for reference by the turquoise lines (top to bottom, respectively). Hundred sets of 10, 30, 40, 50, 60, 80, 100, or 200 cells were randomly sampled. Medians of L/s ratio were calculated for each extracted sample and are plotted. As expected, the dispersion of the medians decreases when increasing the sample size. Medians of the 10-cell samples at a given time point are sometimes found overcrossing the 244-cell median at another time point. Thus, the analysis of only 10 cells in a given experiment could lead to false negative results. **(C)** Statistical significances between exposure times were determined on the same data set as in **(B)** (comprising the 244 original cells as well as the random samples of varying size). Friedman test was used to determine the global significance (upper left graph), Wilcoxon signed-rank test was used when comparing two time points. Data are expressed as –log10 (*pval*). Significance obtained using the 244 cells original set is shown for reference as a blue line. Gray lines represent the lowest value considered as significant [i.e., –log10 (0.05)]. As expected, values increase when increasing sample size. Thirty-cell sample size always produces significant *pval*, except when comparing time 120 min to time 240 min.

However, the above method measures cell shape at experimental end-point and thus does not provide a dynamic view of cell shape changes at an individual level. To circumvent these limitations, we used a lens-free, live-cell imaging device that allows the monitoring of a large number of cells. Images obtained with this system were then analyzed to determine changes in the L/s ratio of individual cells according to treatment. To set up the experimental conditions, MRC5 cells were first seeded in FCS-containing medium and then switched to serum-free medium (Figure S2A). A significant cell elongation was observed 12 h after medium change, a phenomenon that endured for at least 48 h. For all subsequent experiments, treatment was thus applied after 48 h incubation in serum-free medium. Under these conditions, addition of E2 (10^−7^ M) resulted in a significant reduction of L/s ratio, as measured on 244 cells, that was obvious 120 and 240 min after treatment initiation (Figure 2A). We next wanted to determine the minimal number of cells to be measured that would allow to reaching statistical significance. To this end, we randomly sampled 100 sets of 10, 30, 40, 50, 60, 80, 100, or 200 cells from our initial 244-cell data set. Medians of L/s ratio were calculated for each extracted sample. As expected, the dispersion of the medians decreases when increasing the sample size (Figure 2B). Medians of the 10-cell samples at a given time point are sometimes found overcrossing the 244-cell median at another time point. Thus, the analysis of only 10 cells in a given experiment could lead to false negative results. This effect was not observed for samples comprising 30 cells or more.

Statistical significances of the changes observed in the L/s medians were then determined on the same data sets (Figure 2C). Again, data sets comprising 10 cells often failed to reach significance (i.e., *p* < 0.05). In contrast, the use of 30-cell data sets allowed to reaching significance at the global level, i.e., considering all three time points together. This was also the case when comparing time points two-by-two, except for the smaller variations between 120 and 240 min. An additional experiment set with expanded time points showed a continuous reduction of L/s ratio along exposure time (Figure S2B). However, the difference between 180 and 240 min after E2 addition, although statistically significant, was much reduced. This indicates that recording cell shape up to 180 min after treatment initiation is fairly sufficient to observe a statistically significant effect. Under these conditions, supplementation with DMSO, the vehicle used for E2 as well as for all hereafter used compounds, did not impact L/s ratio (Figure S2C). We next tested the reproducibility of our observation. To this end, we performed three independent experiments and observed similar reduction of L/s ratio upon E2 treatment, whereas DMSO had no effect (Figure S2D). Importantly, the L/s values reached after 180 min within each treatment type were not significantly different from one experiment to the other. Altogether, our data show that an E2-induced effect can be reliably evidenced by measuring the L/s ratio of 30 MRC5 cells 180 min after treatment onset.

**Figure 3:**
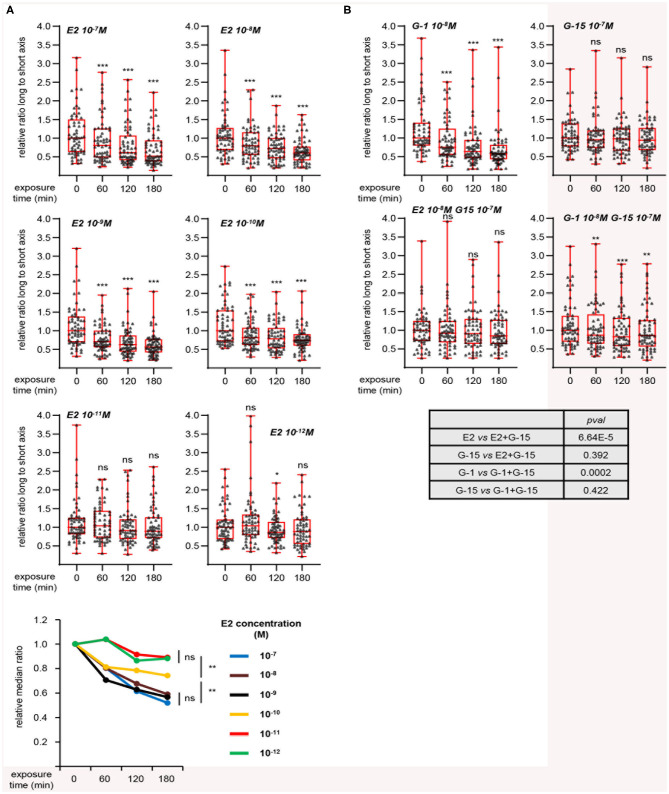
Dynamic measurement of morphological changes induced by E2 in a GPER-dependent manner. **(A)** Cells were treated with the indicated concentration of E2 and individually followed. Images generated with the Cytonote system were analyzed using ImageJ. Ratio long to short axis (L/s) was determined for individual cells followed at the indicated times. Data are expressed relative to the median of L/s ratio at time 0. Graphs represent two pooled experiments, each including 30 cells. Statistical significance relative to time 0 was analyzed using Wilcoxon signed-rank test. Bottom graph summarizes the above data, with the relative medians plotted as a function of time. For this graph, significance at time 180 min was analyzed using Mann-Whitney test. **(B)** Same as above analyzing the effect of G-1 and G-15 (GPER agonist and antagonist, respectively), alone or in combination as indicated. Bottom table displays the significance (estimated by Mann-Whitney tests) of the indicated comparisons. *p < 0.05; **p < 0.005; ***p < 0.0005; ns: non significant.

To further characterize this effect, we investigated the dose-response of L/s ratio to E2 (Figure 3A). A decrease of this ratio was observed for E2 concentrations from 10^−7^ to 10^−10^ M, although the latter dose displayed a moderate effect. Applying 10^−11^ or 10^−12^ M did not induce any change in cell shape. As shown above, MRC5 cells express the GPER membrane estrogen receptor, but not the classical ERα nuclear receptor, suggesting that the effect of E2 may be mediated by GPER. Consistently, treating cells with the GPER synthetic agonist G-1 resulted in an effect similar to that obtained with E2 treatment (Figure 3B). In contrast, the GPER antagonist G-15, by itself innocuous on L/s ratio, completely blocked the reduction of cell elongation exerted by E2. Co-treatment with G-1 and G-15 resulted in a moderate decrease of L/s ratio. However, further statistical analysis showed that this effect was not significantly different from that of G-15 alone. The effect of E2 was also blocked by another GPER synthetic antagonist, G-36 (Figure S3). We conclude that MRC5 cell elongation reflects GPER activation status.

**Figure 4:**
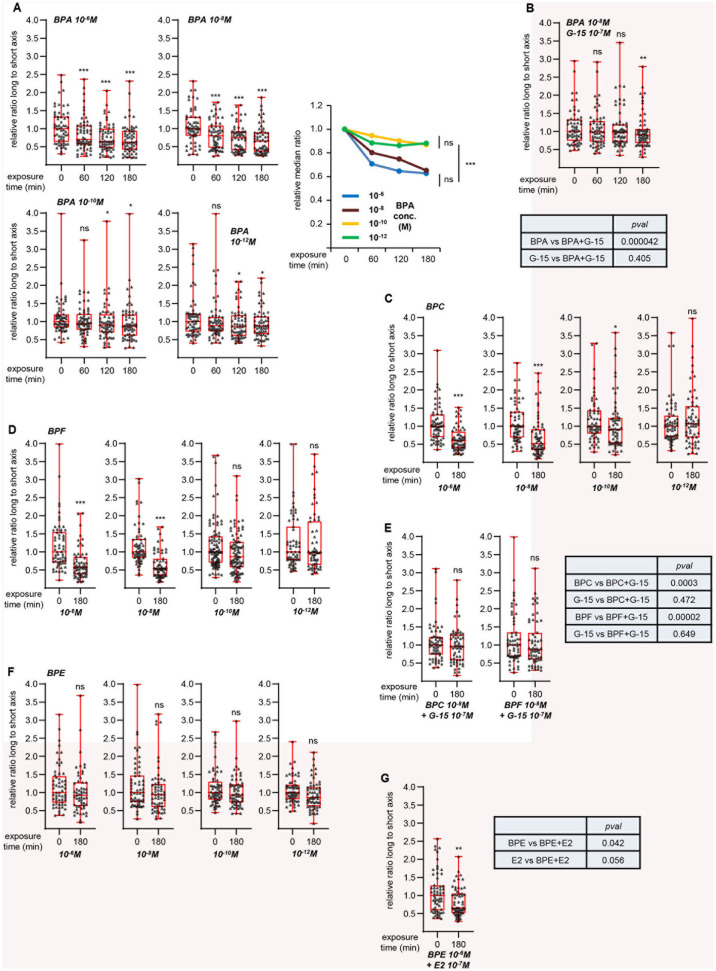
GPER-dependent morphological changes induced by bisphenols. Same approach as Figure 3. **(A)** Effect of the indicated concentrations of bisphenol A (BPA). Right graph summarizes the data, with the relative medians plotted as a function of time. For this graph, significance at time 180 min was analyzed using Mann-Whitney test. **(B)** Effects of G-15 co-treatment on BPA exposure. **(C,D)** Effects of bisphenol C (BPC, **C**) and F (BPF, **D**) at the indicated concentrations. **(E)** Effect of G-15 pretreatment on BPC and BPF. **(F)** Effects of bisphenol E (BPE) at the indicated concentrations. **(G)** Effect of BPE pretreatment on E2 exposure. All graphs represent pools of two independent experiments, each with *n* = 30 cells. Significance relative to time 0 was analyzed using Wilcoxon signed-rank test. *p < 0.05; **p < 0.005; ***p < 0.0005; ns: non significant. Tables displays the significances (estimated by Mann-Whitney tests) of the indicated comparisons.

To further validate this hypothesis, we focused on a synthetic compound, bisphenol A (BPA), reported to activate GPER (22, 23, 27). Cell viability tests indicated that exposure to 10^−5^ M BPA did not significantly impact MRC5 cell survival (Table S2). We then measured L/s ratio after treatment with various BPA concentrations, with 10^−6^ M as the highest (Figure 4A). We observed a time-dependent, dose-dependent reduction of L/s ratio upon BPA exposure. Co-treatment with G-15 partially impaired this effect (Figure 4B). As in the case of G-1 and G-15 co-treatment above, statistical analysis showed that the effect exerted by BPA+G-15 did not significantly differ from that obtained with G-15 alone, indicating that BPA induces morphological changes in a GPER-dependent manner. We next studied the effects of three related bisphenol compounds. BPC and BPF dose-dependently reduced the L/s ratio (Figures 4C,D), an effect that was blocked by co-treatment with G-15 (Figure 4E), indicating that these chemicals activate GPER. In contrast, BPE was inactive at all concentrations tested (Figure 4F). We tested the possibility that BPE could behave as a GPER-antagonist, rather than agonist. However, BPE did not inhibit the effect exerted by E2 (Figure 4G).

The data above indicate that the dynamic measure of L/s ratio is a read-out for agonist or antagonist effects exerted on GPER. This approach could thus be used as a screening method to determine whether a given compound, including EDCs, targets GPER. The effect of agonists would be blocked by co-treatment with G-15, whereas antagonists would inhibit the action of E2. To validate this possibility, we focused on 19 compounds (see characteristics on Table S1), reported to act as EDCs, belonging to different chemical families and with different applications. We first examined their toxicity in MRC5 cells (Table S2). We then evaluated these compounds at three different concentrations for their capacity to impact on L/s ratio. For each compound, the maximal concentration that we used was 10-fold less than the highest non-toxic dose. All data are shown on Figures S4A–S and summarized on Table 1. Four compounds (chlorpyrifos, DEHP, dienochlor and quinoxyfen) induced a dose-dependent reduction of the L/s ratio, to an extent that was comparable to what observed with E2. Co-treatment with G-15 resulted in effects that were *i-* not significantly different from that of G-15 alone and *ii-* different from those observed when using each compound individually (although significance was not reached when comparing DEHP to DEHP+G-15). We concluded that these four chemicals behaved as GPER agonists.

**Table 1:** Effect of EDC exposure on L/s axis in MRC5 cells.

	**Effect at max. conc**.		**Effect with G15**			**Effect with E2**			**Comments**
	**Conc**.	**Rel. med**	**Rel. med**	**Signif. vs**.	**Signif. vs**.	**Rel. med**	**Signif. vs**.	**Signif. vs**.	
		**(% at**	**(% at**	**EDC**	**G15**	**(% at**	**EDC**	**E2**	
		**180 min)**	**180 min)**	**only**	**only**	**180 min)**	**only**	**only**	
Chlorpyrifos	10^−6^ M	56.52	93.15	0.00003	0.419	nd			Agonist
DEHP	10^−6^ M	61.39	94.84	0.0769	0.8479	nd			Trend to agonist
Dienochlor	10^−8^ M	63.37	80.11	0.0145	0.097	nd			Agonist
Quinoxyfen	10^−7^ M	66.29	92.94	0.0056	0.813	nd			Agonist
4-Tert-Octylphenol	10^−7^ M	81.41	nd			69.37	0.0632	0.0381	Weak antagonist
Fenitrothion	10^−6^ M	82.04	nd			75.54	0.854	0.007	Antagonist
Tau-Fluvalinate	10^−7^ M	84.52	nd			55.12	0.0002	0.466	No effect
Bifenthrin	10^−6^ M	84.57	nd			54.73	7.901E-08	0.459	No effect
Chlorpyrifos-methyl	10^−6^ M	85.46	nd			60.99	0.024	0.3186	No effect
Cypermethrin	10^−6^ M	86.01	nd			88.74	0.532	0.0001	Antagonist
Dieldrin	10^−6^ M	86.24	nd			63.28	0.069	0.217	No effect
Ethylparaben	10^−6^ M	86.88	nd			69.94	0.0054	0.1691	No effect
Azoxystrobin	10^−6^ M	87.18	nd			69.32	0.3779	0.0003	Weak antagonist
Malathion	10^−8^ M	87.34	nd			94.06	0.879	0.00004	Antagonist
Imidacloprid	10^−7^ M	88.22	nd			68.07	0.014	0.195	No effect
Methylparaben	10^−7^ M	89.78	nd			59.60	7.382E-06	1.000	No effect
Penconazole	10^−6^ M	90.98	nd			65.29	0.006	0.268	No effect
Deltamethrin	10^−6^ M	91.31	nd			99.56	0.793	0.0001	Antagonist
Piperonyl-Butoxide	10^−7^ M	107.55	nd			63.78	2.929E-07	0.644	No effect
E2	10^−7^ M	51.9	84.77	0.00006	0.392	na			Agonist
G15	10^−7^ M	94.21	na			84.77	0.00006	0.392	Antagonist

*Cells were exposed to the indicated EDC. L/s ratios were determined for individual cells at 0 and 180 min after EDC addition. Results (representing two experiments, each measuring 30 cells) are expressed as the median of L/s ratios at 180 min relative to 0 min. Where indicated, cells were co-treated with G-15 or E2. Significance was estimated using Mann-Whitney test. Only the effect of the maximal concentration is shown for each EDC (see Figures S4A–S for complete data set). Effects observed with E2 or G-15 (complete results on Figure 3) are displayed for reference. na, not applicable; nd, not determined*.

In contrast, when used alone, the other 14 compounds tested here displayed a more moderate effect (or lack thereof) on L/s ratio. Co-treatment with E2 revealed that some of these chemicals behaved as antagonists. For example, the L/s ratios resulting from malathion + E2 co-treatment were strongly different from what obtained with E2 alone, but not different from what observed with malathion alone. Oppositely, compounds such as penconazole did not block the effect of E2, suggesting that they are inactive on GPER.

## Discussion

In this report, we show that E2 induces a dose-dependent, time-dependent morphological change in the MRC5 human fibroblastic cell line. This leads to cell spreading which can be quantified by measuring the ratio between the long and the short cell axes. This effect is analogous to that previously observed in human dermal fibroblasts (hDF) in primary culture (26) as well as in breast cancer cells (28, 29). These actions of E2 do not depend on the classical nuclear estrogen receptors but on GPER, a seven-transmembrane domain estrogen receptor, as well as its downstream effectors ERK1/2. In hDF, this was formally proven by the loss of E2 effects upon shRNA-mediated GPER inactivation. As documented on Figure S1, transient genetic inactivation of GPER is inefficient in MRC5 cells. Stably inactivating the receptor (e.g., using a Crispr-Cas9 approach) is difficult to envision given that the selection procedure would likely exceed the low number of possible cell passages in culture. Pharmacological approaches however show that the effects of E2 rely on GPER. Indeed, this phenomenon can be mimicked by supplementation with the synthetic GPER agonist G-1 and the effects of E2 can be blocked by co-treatment with the GPER antagonists G-15 and G-36.

The work presented here points to the possibility of a general method to screen compounds for their capacity to signal through GPER. Cell fixation and staining are not required, enabling a dynamic monitoring of individual cells along treatment. Furthermore, the use of the Cytonote lens-free device allows to visualizing large fields and thus a large number of cells with rapid image acquisition. Our statistical analyses show that considering as few as 30 cells for 3 h is enough to reach significance. The method used here appears very sensitive and, in some cases, statistical tests can demonstrate that very small changes in the L/s ratio are strongly significant at the population level. However, the relevance of these small variations can be questioned, in particular when no relation between dose and response is observed. For instance, penconazole exerts a 10% reduction of the L/s ratio at 10^−8^ M (with *pval*: 0.008), but not at 10^−6^ or 10^−10^ M (see Figure S4Q). Similarly, dieldrin exerts a 12–15% reduction of the relative L/s ratio with *pval* < 0.05 at all three concentrations tested (i.e., without dose-response relations; see Figure S4K). Noteworthy, exposure to 10^−12^ M BPA also results in a 12% reduction of the L/s ratio (with *p* ~ 0.01; Figure 4A). However, similarly to E2, BPA displays a clear dose-dependent effect with a strong reduction of the L/s ratio at maximal concentration (~40% at 10^−6^ M). It thus cannot be excluded that small variations in L/s ratio reflect experimental noise rather than relevant signal. With such considerations in mind, it appears sensible to consider only the compounds that induce large variations (i.e., within the range of those observed upon E2 exposure) in L/s ratio as GPER agonists. Altogether, the GPER-specific method described here appears easy to set up, robust and cheap.

As a proof-of-concept, we have performed a low throughput screen in which 23 synthetic compounds were examined for their capacity to modulate GPER activation. To the best of our knowledge, most of these compounds have not been reported for an effect on GPER (or lack thereof). Seven compounds (bisphenols A, C and F, chlorpyrifos, DEHP, dienochlor and quinoxyfen) were found to display agonist activities, according to the above criterion. In support to this view, co-treatment with G-15 abolished the cellular effect elicited by these chemicals. In contrast, 16 compounds appeared inactive on cell morphology when used alone. The capacity of six of these chemicals to block the activities of E2 allows to consider them as GPER-antagonists, whereas the remaining 11 do not appear to exert any effect on GPER. Remarkably, structurally related compounds do not necessarily fall into the same category. Consistent with the current literature, BPA acted as a GPER agonist (22, 23, 27), as did BPC and BPF. In contrast, BPE, which only differs from BPF and BPA by the presence or absence of a methyl group (respectively), was completely inactive on GPER. In this line, we also observed that chlorpyrifos and chlorpyrifos-methyl displayed different behaviors (agonist and inactive, respectively) toward GPER. In contrast, the related compounds cypermethrin and deltamethrin both acted as GPER antagonists. Structural studies will be required to determine the bases of these differences and similarities. Our work identified EDCs that positively or negatively modulate the activities of GPER in normal human fibroblasts. On another hand, activation of GPER in breast cancer-associated fibroblasts (CAFs) promotes cancer progression (30–32). Whether the EDCs identified here also modulate GPER activities in CAFs remains to be investigated, as well as the consequences of these possible regulations.

In summary, we propose our approach as a potential screening method to determine whether a given compound agonizes or antagonizes GPER. Of note, an effect observed here of GPER does not exclude possible actions on other receptors, such as ERα or AR. Furthermore, the present assay is purely cell-based and cannot be used to predict the effects of chemicals *in vivo*.

## Data Availability Statement

All datasets generated for this study are included in the article/Supplementary Material.

## Author Contributions

SP, CF, and VT: performed experiments. CC: performed experiments and analyzed data. J-MV: planned experiments, analyzed data, and wrote the paper. All authors contributed to the article and approved the submitted version.

## Conflict of Interest

The authors declare that the research was conducted in the absence of any commercial or financial relationships that could be construed as a potential conflict of interest.

## Supplementary Material

Figure S1 Analysis of MRC5 cells. **(A)** MRC5 cells were cultured in the presence of untreated (FCS), or desteroidated serum-containing medium supplemented with vehicle (DS) or 10^−7^ M E2. Proliferation is shown relative to day 0. Values are the mean of two independent experiments performed in triplicate with error bars representing SEM. Significance (relative to day 0) was analyzed using Student *t*-test. ^***^*p* < 0.0005; ns: non significant. Graph on the right zooms the lower part of the left graph. **(B)** Expression of GPER mRNA (left) and protein (right) 72 h after transfection with the indicated siRNA. Left: analysis was performed by real-time PCR. Data are presented relative to siControl-treated samples and are the average of two independent experiments performed in triplicate. Error bars represent SEM. Significance (relative to siC) was analyzed using Student *t*-test. ^*^*p* < 0.05; ^**^*p* < 0.005; ns: non significant. Right: expression of the indicated proteins after transfection with the indicated siRNA. Hsp90 was used as a loading control.

Figure S2 Validation of the Cytonote approach in MRC5 cells. **(A)** Cells were seeded in untreated serum-containing medium (FCS) then switched to DMEM medium in the absence of serum. Graph represents a single experiment with *n* = 30. **(B)** Cells were treated with 10^−7^ M E2. Graph represents a pool of two independent experiments, each with *n* = 30. **(C)** Cells were treated with 1/1,000 (vol/vol) DMSO. Graph represents a pool of two independent experiments, each with *n* = 30. **(D)** Cells were treated with DMSO (1/1,000 vol/vol) or 10^−7^ M E2. Each graph represent a single experiment with *n* = 30. Lower graph presents the relative median at 180 min (expressed as % from time 0) for each experiment. Global *pval* comparing all DMSO or all E2 treatment was calculated using Kruskal-Wallis test. Data on graphs are expressed relative to the median of the L/s ratio at time 0. Statistical significance was analyzed using Wilcoxon signed-rank test. ^*^*p* < 0.05; ^**^*p* < 0.005; ^***^*p* < 0.0005; ns: non significant.

Figure S3 Antagonistic activities of G-36. Cells were treated with E2 and/or the GPER antagonist G-36 and analyzed at the indicated time. Statistical significance was determined using Wilcoxon signed-rank test. ^*^*p* < 0.05; ^**^*p* < 0.005; ^***^*p* < 0.0005; ns: non significant. Tables displays the significances (estimated by Mann-Whitney tests) of the indicated comparisons.

Figure S4 Morphological changes induced by endocrine disrupting compounds on MRC5 cells. Cells were treated with the indicated compounds at the various concentrations. **(A–D)** Compounds, significantly inducing a morphological change in MRC5, were also used in combination with the GPER antagonist G-15. **(E–S)** Compounds that did not significantly induce any morphological change were also used in combination with E2. Data are also summarized on Table 1. All graphs represent two pooled independent experiments, each with *n* = 30. Statistical significance was determined using Wilcoxon signed-rank test. ^*^*p* < 0.05; ^**^*p* < 0.005; ^***^*p* < 0.0005; ns: non significant.

Table S1 Characteristics of the compounds used in this study.

Table S2 Evaluation of cell viability after exposure to the compounds used in this study. Cell number was estimated after 48 h treatment with the indicated compound and expressed relative (%) to treatment with vehicle (DMSO). Results represent mean of two experiments performed in triplicate and are expressed relative (%) to treatment with vehicle ± s.e.m. Significance was estimated used Student t-test. ^*^*p* < 0.05; ^**^*p* < 0.01; ^***^*p* < 0.005; ns: not significant. nd: not determined.
