# Supplementary material for: A Cell-Based Method to Detect Agonist and Antagonist Activities of Endocrine-Disrupting Chemicals on GPER
Source: Front Endocrinol (Lausanne). 2020 Aug 14;11:547. doi: 10.3389/fendo.2020.00547 (PMC7456940; doi:10.3389/fendo.2020.00547)
Supplement: Figure S1 — Analysis of MRC5 cells. (A) MRC5 cells were cultured in the presence of untreated (FCS), or desteroidated serum-containing medium supplemented with vehicle (DS) or 10−7 M E2. Proliferation is shown relative to day 0. Values are the mean of two independent experiments performed in triplicate with error bars representing SEM. Significance (relative to day 0) was analyzed using Student t-test. ***p < 0.0005; ns: non significant. Graph on the right zooms the lower part of the left graph. (B) Expression of GPER mRNA (left) and protein (right) 72 h after transfection with the indicated siRNA. Left: analysis was performed by real-time PCR. Data are presented relative to siControl-treated samples and are the average of two independent experiments performed in triplicate. Error bars represent SEM. Significance (relative to siC) was analyzed using Student t-test. *p < 0.05; **p < 0.005; ns: non significant. Right: expression of the indicated proteins after transfection with the indicated siRNA. Hsp90 was used as a loading control. [file Data_Sheet_1.PDF]

A

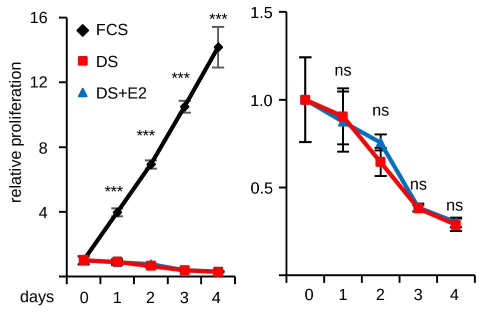

B

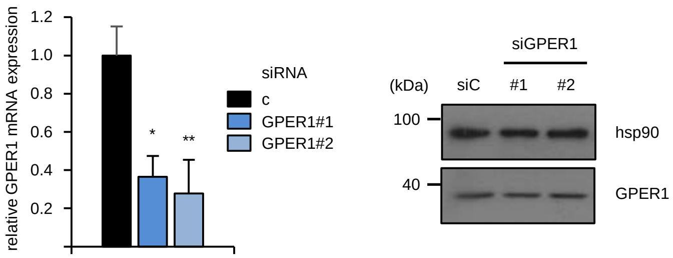

Périan et al., Figure S1

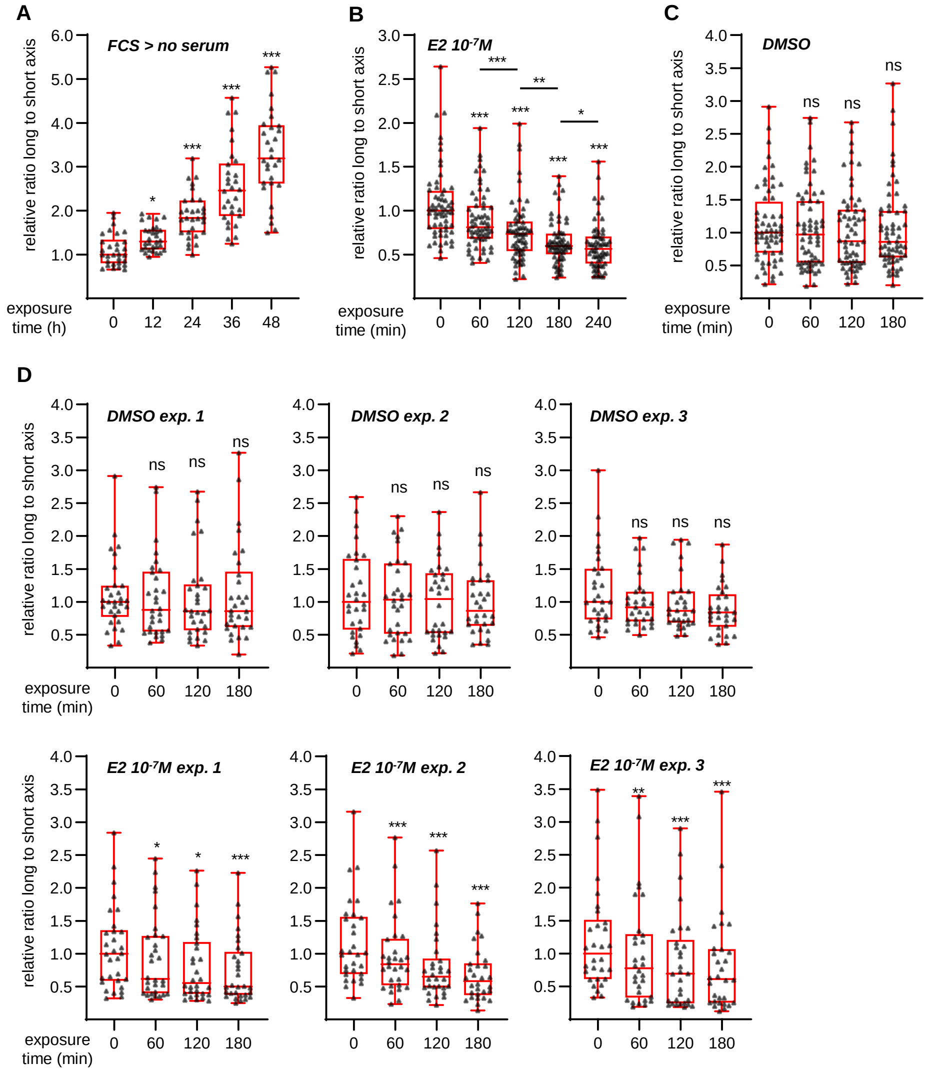

	relative median at 180 min (%)			global *pval*
	exp. 1	exp. 2	exp. 3	
DMSO	86.03	86.28	84.28	0.7937
E2	50.07	58.55	61.44	0.9352

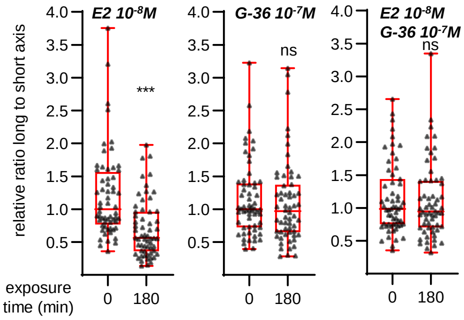

	pval
E2 *vs* E2+G-36	1.090E-5
G-36 *vs* E2+G-36	0.801

Périan et al., Figure S3

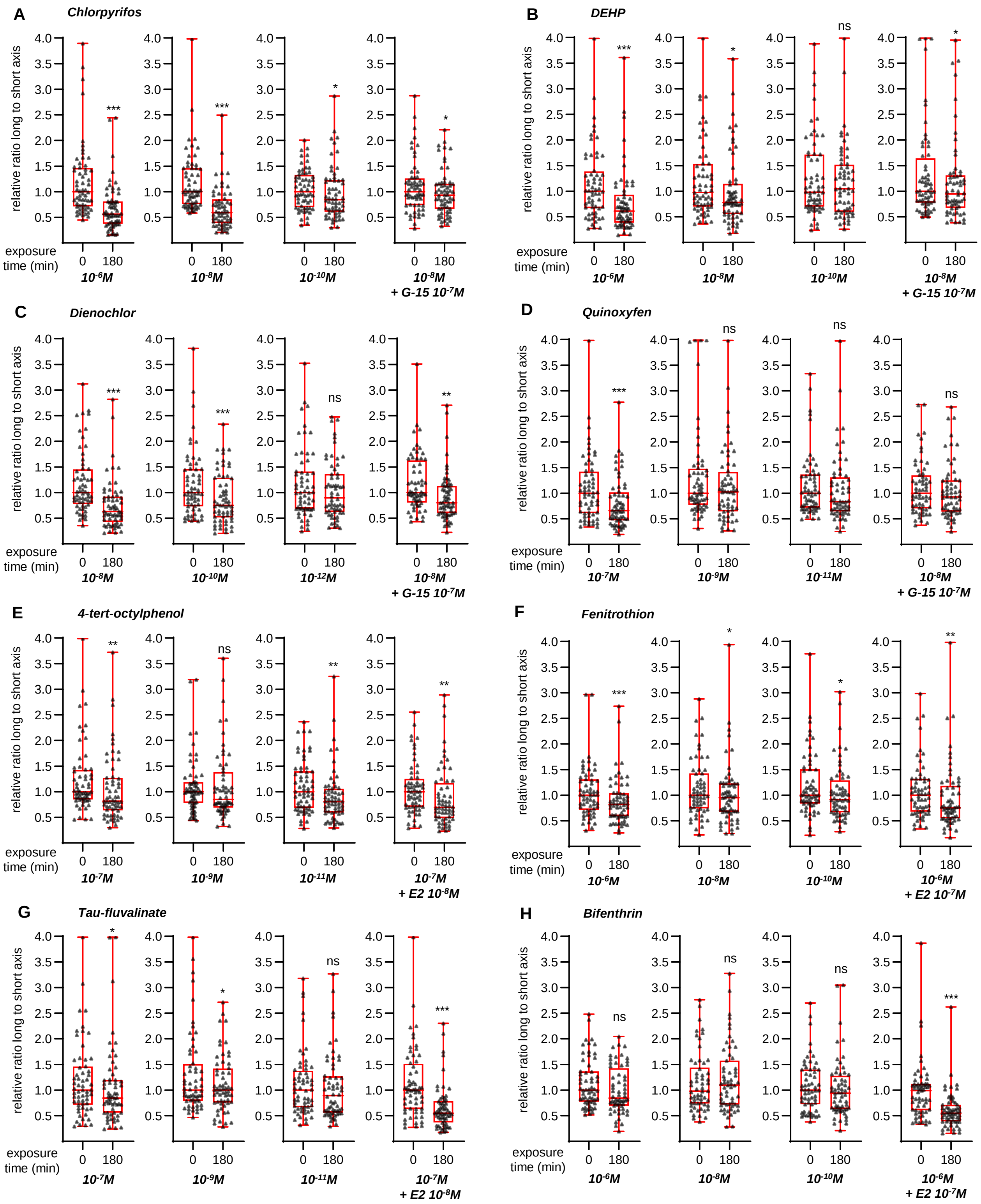
A
Chlorpyrifos
B
DEHP
C
Dienochlor
D
Quinoxyfen
E
4-tert-octylphenol
F
Fenitrothion
G
Tau-fluvalinate
H
Bifenthrin
relative ratio long to short axis
exposure time (min)
0
180
10-6M
10-8M
10-10M
10-12M
10-7M
10-9M
10-11M
+ G-15 10-7M
+ E2 10-8M
+ E2 10-7M
ns
*
**
***

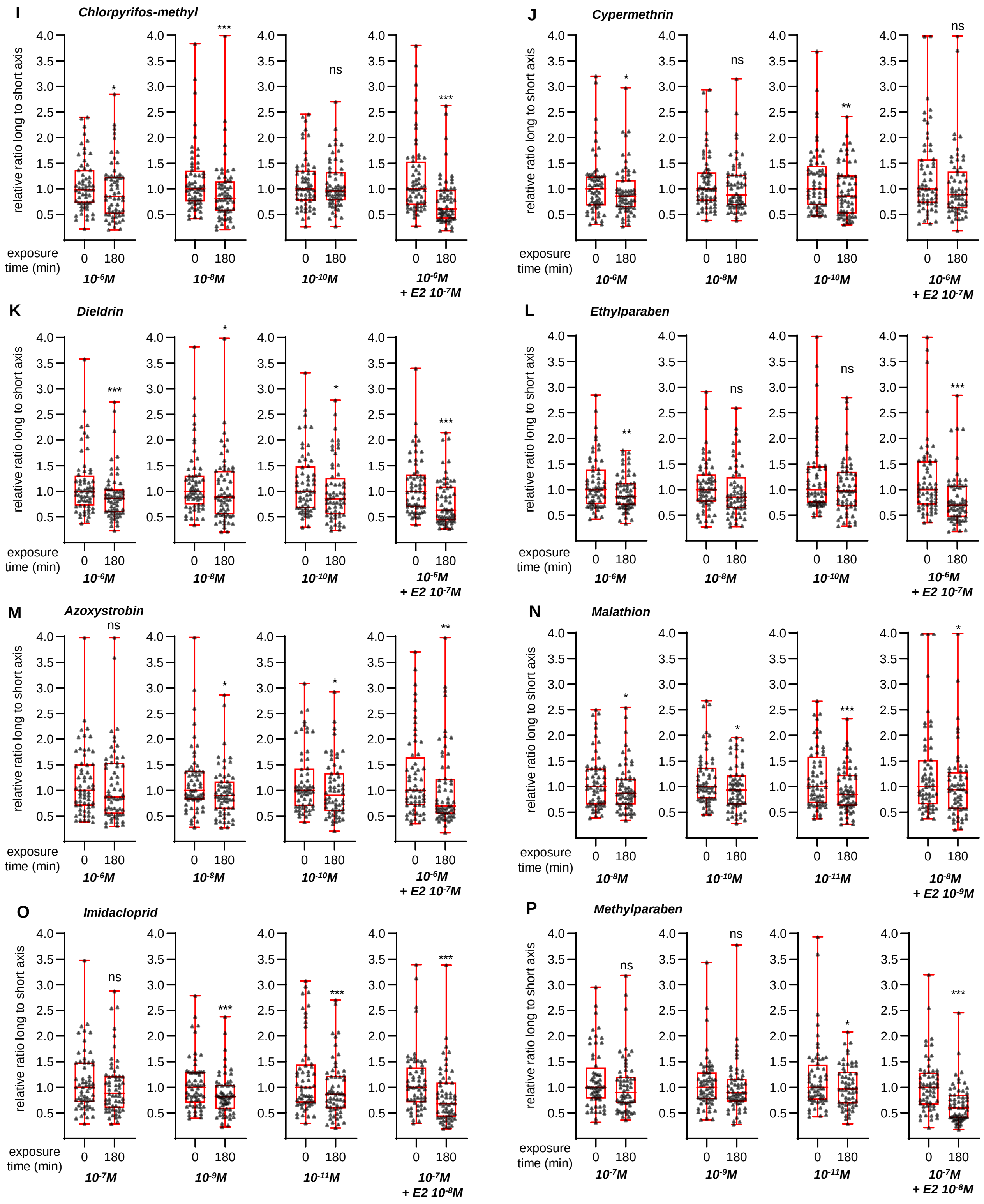

Périan et al., Figure S4 continued

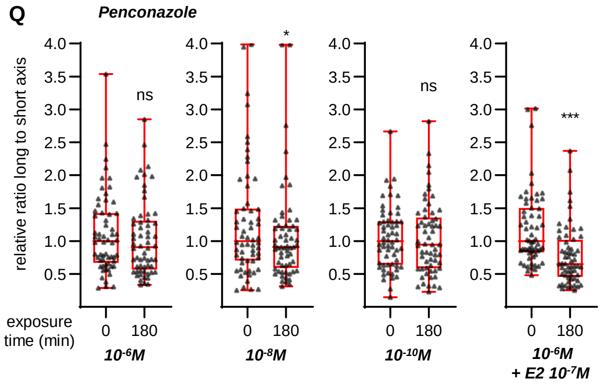
Q
Penconazole
relative ratio long to short axis
4.0
3.5
3.0
2.5
2.0
1.5
1.0
0.5
ns
*
ns
***
exposure time (min)
0
180
10-6M
10-8M
10-10M
10-6M + E2 10-7M

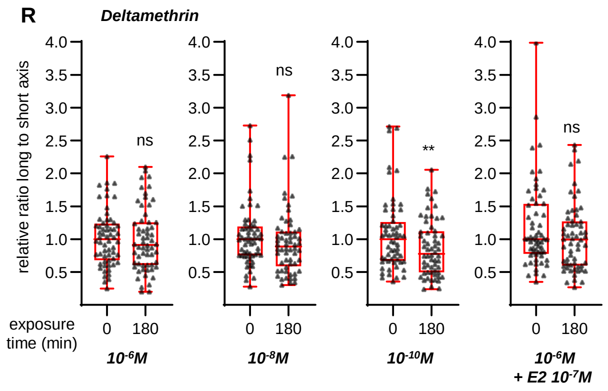
R
Deltamethrin
relative ratio long to short axis
4.0
3.5
3.0
2.5
2.0
1.5
1.0
0.5
ns
ns
**
ns
exposure time (min)
0
180
10-6M
10-8M
10-10M
10-6M + E2 10-7M

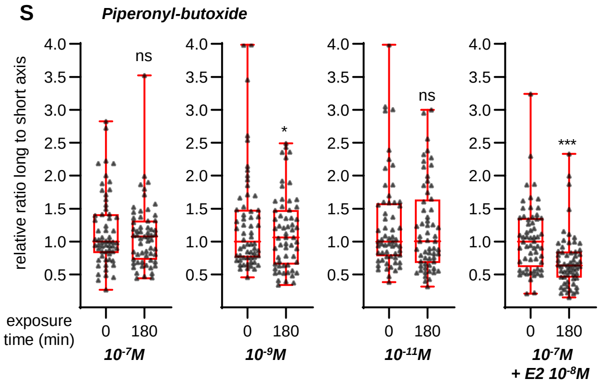
S
Piperonyl-butoxide
relative ratio long to short axis
4.0
3.5
3.0
2.5
2.0
1.5
1.0
0.5
ns
*
ns
***
exposure time (min)
0
180
10-7M
10-9M
10-11M
10-7M + E2 10-8M

Table S1: Characteristics of the compounds used in this study

	Class	Use	Formula	CAS nb	MW	Manufacturer	Catalog reference
**Azoxystrobin**	Strobilurine	Fongicide	$C_{22}H_{17}N_3O_5$	131860-33-8	403,39	Sigma	31697
**Bifenthrin**	Pyrethroid	Insecticide	$C_{23}H_{22}ClF_3O_2$	82657-04-3	422,87	Sigma	34314
**Chlorpyrifos**	Organophosphate	Insecticide	$C_9H_{11}Cl_3NO_3PS$	2921-88-2	350,59	Sigma	45395
**Chlorpyrifos-méthyl**	Organophosphate	Insecticide	$C_7H_7Cl_3NO_3PS$	5598-13-0	322,53	Sigma	45396
**Cypermethrin**	Pyrethroid	Insecticide	$C_{22}H_{19}Cl_2NO_3$	52315-07-8	416,3	Sigma	36128
**Deltamethrin**	Pyrethroid	Insecticide	$C_{22}H_{19}Br_2NO_3$	52918-63-5	505,2	Sigma	45423
**Dienochlor**	Organochloride	Insecticide	$C_{10}Cl_{10}$	2227-17-0	474,64	Sigma	45443
**Dieldrin**	Organochloride	Insecticide	$C_{12}H_8Cl_6O$	60-57-1	380,91	Sigma	33491
**Fenitrothion**	Organophosphate	Insecticide	$C_9H_{12}NO_5PS$	122-14-5	277,23	Sigma	45487
**Imidacloprid**	Neonicotinoid	Insecticide	$C_9H_{10}ClN_5O_2$	138261-41-3	255,66	Sigma	37894
**Malathion**	Organophosphate	Insecticide	$C_{10}H_{19}O_6PS_2$	121-75-5	330,36	Sigma	36143
**Penconazole**	Triazole	Fongicide	$C_{13}H_{15}Cl_2N_3$	66246-88-6	284,18	Sigma	36189
**Piperonyl-Butoxide**	other	Insecticide	$C_{19}H_{30}O_5$	51-03-6	338,44	Sigma	45626
**Quinoxyfen**	Quinoline	Pesticide	$C_{15}H_8Cl_2FNO$	124495-18-7	308,13	Sigma	46439
**Tau-Fluvalinate**	Pyrethroid	Insecticide	$C_{26}H_{22}ClF_3N_2O_3$	102851-06-9	502,91	Sigma	46294
**Methylparaben**	Paraben	Cosmetics	$C_8H_8O_3$	99-76-3	152,15	Sigma	PHR 1012-1G
**Ethylparaben**	Paraben	Cosmetics	$C_9H_{10}O_3$	120-47-8	166,17	Sigma	PHR1011-1G
**Bisphenol A**	Bisphenol	Plastics	$C_{15}H_{16}O_2$	80-05-7	228,29	Sigma	239658
**Bisphenol C**	Bisphenol	Plastics	$C_{17}H_{20}O_2$	79-97-0	256,34	Sigma	68118
**Bisphenol E**	Bisphenol	Plastics	$C_{14}H_{12}O_2$	2081-08-5	214,26	Sigma	4487
**Bisphenol F**	Bisphenol	Plastics	$C_{13}H_{12}O_2$	620-92-8	200,23	Sigma	51453
**DEHP**	Phtalate	Plastics	$C_{24}H_{38}O_4$	117-81-7	390,6	Sigma	36735
**4-Tert-Octylphenol**	AlkylPhenol	Paint	$C_{14}H_{22}O$	140-66-9	206,33	Sigma	290823

Table S2: Evaluation of cell viability after exposure to the compounds used in this study.

Cell number was estimated after 48 h treatment with the indicated compound and expressed relative (%) to treatment with vehicle (DMSO). Results represent mean of two experiments performed in triplicate and are expressed relative (%) to treatment with vehicle +/- s.e.m. Significance was estimated used Student t-test. *: *p* <0.05; **: *p* <0.01; ***: *p* <0.005; ns: not significant. nd: not determined

	10^{-4} M	10^{-5} M	10^{-6} M	10^{-7} M	10^{-8} M
**Bisphenol A**	170.9 +/- 10.2 ***	109.8 +/- 1.6 ns	112.0 +/- 10.0	nd	nd
**Bisphenol C**	175.6 +/- 11.2 ***	115.7 +/- 4.1 ns	118.0 +/- 3.9	nd	nd
**Bisphenol E**	221.7 +/- 32.9 *	134.1 +/- 19.9 ns	140.4 +/- 6.4	nd	nd
**Bisphenol F**	188.8 +/- 66,5 ns	116.3 +/- 23.4 ns	107.3 +/- 20.8	nd	nd
**Azoxystrobin**	139.4 +/- 7.8 ***	114.6 +/- 6.9 ns	103.5 +/- 2.8	nd	nd
**Bifenthrin**	110.9 +/- 6.5 ns	82.3 +/- 9.3 *	101.9 +/- 3.1	nd	nd
**Chlorpyrifos**	78.4 +/- 28.5 ns	82.6 +/- 6.7 ns	92.7 +/- 8.1	nd	nd
**Chlorpyrifos-méthyl**	58.2 +/- 47.9 ns	88.1 +/- 3.1 ns	93.5 +/- 11.1	nd	nd
**Cypermethrin**	97.7 +/- 2.8 *	93.1 +/- 6.5 ns	103.7 +/- 1.6	nd	nd
**Deltamethrin**	116.7 +/- 14.7 ns	97.8 +/- 9.9 ns	108.8 +/- 6.6	nd	nd
**Dienochlor**	0.2 +/- 0.1 ***	13.3 +/- 22.7 ***	76.4 +/- 50.5 ns	43.7 +/- 1.7 ***	92.5 +/- 7.5
**Dieldrin**	115.1 +/- 7.4 *	91.1 +/- 8.7 ns	95.1 +/- 8.2	nd	nd
**Fenitrothion**	124.2 +/- 7.2 ns	114.1 +/- 9.4 ns	120.6 +/- 11.2	nd	nd
**Imidacloprid**	168.6 +/- 21.0 *	79.2 +/- 21.2 ns	103.4 +/- 27.5	nd	nd
**Malathion**	77. 5 +/- 7.1 ***	39.0 +/- 7.1 ***	78.4 +/- 9.4 ***	98.6 +/- 6.9 *	119.3 +/- 12.1
**Penconazole**	17.5 +/- 4.6 ***	86.1 +/- 18.4 ns	92.9 +/- 13.9	nd	nd
**Piperonyl-Butoxide**	21. 8 +/- 7.1 ***	103.8 +/- 3.8 *	127. 9 +/- 6.3	nd	nd
**Quinoxyfen**	12.1 +/- 2.7 *	56.2 +/- 9.6 ns	92.3 +/- 33.7	nd	nd
**Tau-Fluvalinate**	279.9 +/- 161, 7 ns	89.2 +/- 6.4 *	155.8 +/- 49.9	nd	nd
**Methylparaben**	126.4 +/- 33.1 ns	73.9 +/- 10.4 *	105.4 +/- 13.6 ns	119.7 +/- 27.2	nd
**Ethylparaben**	230.2 +/- 26.9 ***	112.7 +/- 8.9 ns	110.4 +/- 10.7	nd	nd
**DEHP**	72.4 +/- 9.1 ns	127.5 +/- 35.4 ns	77.9 +/- 21.8	nd	nd
**4-Tert-Octylphenol**	2.01 +/- 1.2 ***	150,8 +/- 10.5 ns	132,6 +/- 25.6	nd	nd
